# The Coexistence of Colorectal Polyps in the Right Colon Increases the Malignant Risk of Laterally Spreading Tumors

**DOI:** 10.1155/2020/3180420

**Published:** 2020-04-14

**Authors:** Xiaonan Shen, Yao Zhang, Yunjia Zhao, Xiaobo Li, Zhizheng Ge, Hua Xiong, Danfeng Sun, Qinyan Gao, Yun Cui, Xiaoyu Chen, Yingxuan Chen, Jingyuan Fang

**Affiliations:** Division of Gastroenterology and Hepatology, Shanghai Institute of Digestive Disease, Renji Hospital, School of Medicine, Shanghai Jiao Tong University Key Laboratory of Gastroenterology and Hepatology, Ministry of Health, 145 Middle Shandong Road, Shanghai 200001, China

## Abstract

**Background:**

The coexistence of colorectal polyps with laterally spreading tumors (LSTs) is commonly observed during colonoscopy. However, there are rare studies that assess the malignant risks for LSTs with colorectal polyps, which might largely contribute to further strategies of treatment and follow-up plans in LSTs.

**Methods:**

We conducted a retrospective cohort study that enrolled 206 patients with LSTs in the Endoscopy Center and Endoscopy Research Institute, Renji Hospital, Shanghai Jiao Tong University, China. The subjects with LSTs were divided into two groups: the nonpolyp group with 89 patients and the polyp group with 117 patients. Binary logistic regression was used to identify the independent predictors of outcomes of interest.

**Results:**

The risk of the polyps' coexistence phenomenon increased in males compared with females (OR = 2.138, *p* = 0.047), especially in those between 50 and 75 years old (OR = 7.074, *p* = 0.036). Tumor size (3–4 cm), LSTs with tubulovillous types, and history of polyps statistically increased the risk of the polyp coexistence phenomenon (OR = 5.768, *p* = 0.003; OR = 36.345, *p* = 0.024; OR = 13.245, *p* < 0.0001, respectively). LST-NG-PD (OR = 20.982, *p* = 0.017) and LSTs ≥ 5 cm (OR = 37.604, *p* = 0.038) notably increased the malignant risk of LSTs. When the simultaneous polyps are located in the right colon, the risk of malignant LSTs (OR = 58.540, *p* = 0.013) positively increased.

**Conclusion:**

The simultaneous colorectal polyps in the right colon were the most important risk factor to predict the malignant risk of LSTs.

## 1. Introduction

**Figure 1:**
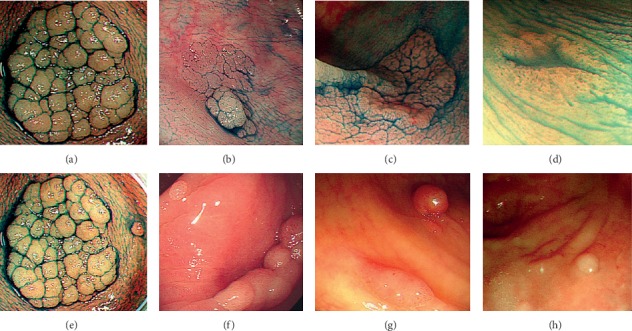
Four subtypes of LSTs with or without polyps. (a) A granular homogeneous LST (LST-G-H). (b) A granular, nodular, and mixed LST (LST-G-M). (c) A nongranular, flat, and elevated LST (LST-NG-F). (d) A nongranular, pseudodepressed LST (LST-NG-PD). (e) A LST-G-H with a polyp. (f) A LST-G-M with a polyp. (g) A LST-NG-F with a polyp. (h) A LST-NG-PD with a polyp.

Large, flat-appearing neoplasms, also known as laterally spreading tumors (LSTs), are at least 10 mm in size and are characterized by horizontally extending growth patterns [1]. LSTs constitute an important contributor to postcolonoscopy colorectal cancer [2]. LSTs are categorized into two types based on colonoscopy: LST-Gs with nodules or granules distributed evenly or not on the surface of the lesion and LST-NGs with a smooth surface without nodules or granules [1]. LST-Gs are further classified into homogeneous (LST-G-H) and nodular mixed (LST-G-M) subtypes, and LST-NGs comprise flat-elevated (LST-NG-FE) and pseudodepressed (LST-NG-PD) subtypes (Figures 1(a)–1(d)). Two subtypes of LSTs differ not only morphologically but also in clinicopathologic features. The carcinoma incidence rate and the submucosal invasion rate were reported to be higher in LST-NG than in LST-G, particularly in LST-NG-PD [3]. Colorectal endoscopic submucosal dissection (ESD), endoscopic mucosal resection (EMR), and endoscopic mucosal resection with precutting (EMRP) are selected to resect LSTs according to the size and pathological features [4–8]. If superficial submucosal invasion (SMI) is suspected, en bloc resection should be the therapy of choice. En bloc resection for superficial neoplasms larger than 20  mm can be achieved by ESD [9].

During the colonoscopy or treatment of LSTs, the coexistence of LSTs and colorectal polyps is common in the same patient (Figures 1(e)–1(h)). Nonetheless, little is known about the risk factors and the relationship with advanced histology regarding the coexistence phenomenon, which are important for providing appropriate treatment and follow-up strategies for patients with LSTs. Here, we evaluated the clinicopathologic differences between LSTs and the coexistence of LSTs and colorectal polyps to determine risk features of the coexistence phenomenon and its relationship with malignant LSTs.

## 2. Methods

This retrospective cohort study enrolled 226 patients who had undergone colonic ESD, EMR, or EMRP for LSTs at the Endoscopy Center and Endoscopy Research Institute, Renji Hospital, Shanghai Jiao Tong University from June 2013 to September 2018, which accounts from most of LST patients. All lesions had been initially identified and referred by well-experienced endoscopists. The inclusion criteria for the subjects were as follows: (1) age ≥ 18 years and (2) LSTs ≥ 10 mm in diameter located in the colorectum, according to the endoscopic presentations. We excluded 20 patients with incomplete clinicopathological data. The included patients were categorized into two groups according to whether LSTs and colorectal polyps existed simultaneously: the nonpolyp group with 87 LST patients and the polyp group with 119 patients. The polyp group was further classified into two groups: nonmalignant LSTs with 94 patients and malignant LSTs with 25 patients. The average of the adenoma detection rate (ADR) of the institution is almost 19%. We collected data on tumor diameter, morphology, location, granular type, and degree of dysplasia. In addition, we collected patients' gender, age, and history of colorectal polyps. The colonoscopies for detecting LSTs were all initial. However, in terms of patients' regular colonoscopy, the ratio of the initial colonoscopy was about 24%. The study was approved by the ethical review board. Name and date approval granted by the ethical board are included in the manuscript. All the participants provided written consent. The study protocol complied with the Declaration of Helsinki.

### 2.1. Endoscopic Criteria of LSTs

All patients were examined using video colonoscopies (Olympus CF-240I or CF-H260; Olympus, Tokyo, Japan). LSTs were subclassified using the endoscopic Kudo classification [10] into LST-Gs and LST-NGs. LST-Gs were further divided into LST-G-H and LST-G-M lesions, and LST-NGs were divided into LST-NG-FE and LST-NG-PD lesions. The locations of LSTs were categorized into the ileocecal valve, ascending colon, hepatic flexure, right transverse colon, left transverse colon, splenic flexure, descending colon, sigmoid colon, and rectum. The right-side colon includes the cecum, ascending colon, hepatic flexure, and two-thirds of the transverse colon. The left-side colon includes the other one-third of the transverse colon, splenic flexure, descending colon, and sigmoid colon.

### 2.2. Histopathological Assessment

We used the World Health Organization (WHO) classification for histopathology [11].

The histological type of LSTs includes the hyperplastic type, low-grade type, high-grade type, and carcinoma. Pathomorphism includes the hyperplastic type, tubular type, tubulovillous type, and serrated adenoma type. In our study, high-grade intraepithelial neoplasm and submucosal invasive carcinoma were defined as malignant types. Hyperplastic neoplasm and low-grade intraepithelial neoplasm were defined as nonmalignant types. Most of the simultaneous colorectal polyps presented small size (<1.0 cm) and hyperplastic features during NBI. These polyps were resected without a histology test. Some of them presented an adenoma type and were resected with further histology test. Those with a histology test showed nonmalignancy. Thus, the histology information of the polyps was incomplete.

### 2.3. Statistical Analysis

SPSS statistical software (version 23.0, IBM Corp, Armonk, NY, 2012) was used to analyze the data. All analyses were exploratory, and two-tailed tests with a significance level of 5% were used throughout. The Pearson chi-square or Fisher's exact test was used to test for the association between categorical variables and outcomes. Binary logistic regression was used to identify the independent predictors of outcomes of interest. Candidate variables with *p* values for association that were less than 0.05 were considered potential risk factors in binary logistic regression analyses. Odds ratios (ORs) with 95% confidence intervals (CIs) from the model were used to quantify the extent of this association.

## 3. Results

### 3.1. The Baseline Clinicopathological Factors of the Enrolled Subjects

A total of 226 patients were treated by ESD, EMR, or EMRP for colorectal LSTs between June 2013 and September 2018 at the Endoscopy Center and Endoscopy Research Institute, Renji Hospital, Shanghai Jiao Tong University. A total of 206 patients were included in this retrospective cohort, of which 20 patients were excluded for the incomplete clinicopathological data. The baseline clinicopathological characteristics of the enrolled subjects are described in Table 1. These patients were divided into two groups: the nonpolyp group with 87 patients and the polyp group with 119 patients. No significant differences were observed in location, morphological type, and degree of dysplasia between the two groups. Statistically, patients ≥ 50 years old and males (*p* = 0.005) were more frequent in the polyp group; 53.4% of LSTs were located in the right colon, whereas 30.6% were in the left colon. LST-G-Ms (44.7%) accounted for the most LSTs, and there were more LST-NGs and LSTs with advanced histology in the polyp group than in the nonpolyp group. The tumor size (*p* = 0.0013) and pathological morphology of LSTs (*p* = 0.0132) were significantly different in the two groups. In our retrospective study, those LSTs comprised four pathological morphologies, including the hyperplastic type, tubular type, tubulovillous type, and serrated adenoma type. The tubular type (71.8%) was the most common in all pathological morphologies. Regarding the history of polyps, 83.8% of patients with a history of polyps presented with the coexistence of LSTs and colorectal polyps (*p* < 0.0001).

### 3.2. Gender, Age, History of Polyps, Morphological Type, and Tumor Size of LSTs Were Associated with the Colorectal Polyp Coexistence Phenomenon

**Figure 2:**
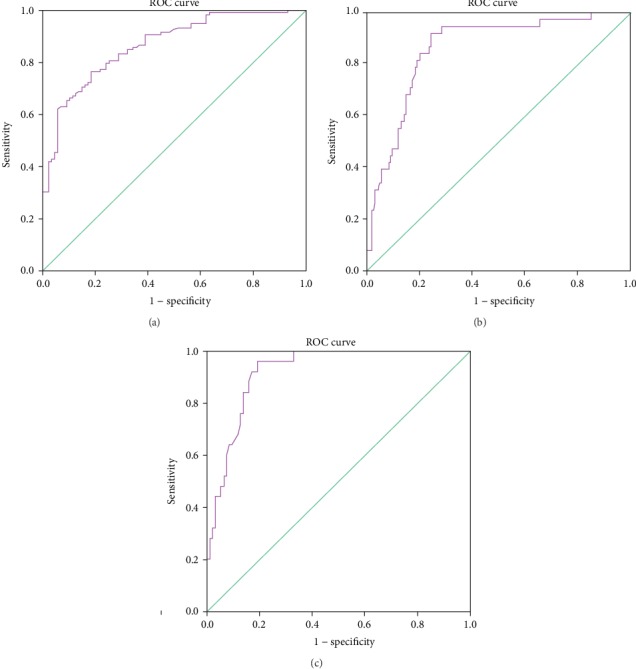
Receiver operating characteristic curve of a binary logistic regression model. (a) Factors associated with the coexistence of LSTs and colorectal polyps included males aged 50-75years, tumor size (3–4 cm), tubulovillous type, and history of polyps. The AUC was 0.866 (95% CI 0.817-0.914; *p* < 0.001). (b) Factors associated with malignant LSTs in enrolled subjects included LSTs ≥ 4 cm and LSTs located in the hepatic flexure and descending colon. The AUC was 0.866 (95% CI 0.804-0.927; *p* < 0.001). (c) Factors associated with malignant LSTs in the polyp group included simultaneous polyps located in the right colon, LST-NG-PD, and LSTs ≥ 5 cm. The AUC was 0.921 (95% CI 0.874-0.969; *p* < 0.001).

To fully demonstrate the factors associated with the coexistence of LSTs and colorectal polyps, we used the binary logistic regression model.  Table 2 presents the best-fitting binary logistic regression model for factors associated with the coexistence of LSTs and colorectal polyps. The risk of the coexistence phenomenon increased in males compared to females (OR = 2.138; 95% CI 1.009–4.530; *p* = 0.047), especially in those aged between 50 years and 75 years (OR = 7.074; 95% CI 1.138–43.981; *p* = 0.036). Tumor size (3–4 cm) statistically increased the risk of LSTs with polyps (OR = 5.768; 95% CI 1.808–18.398; *p* = 0.003). History of polyps was significantly related to the coexistence of LSTs and colorectal polyps (OR = 13.245, *p* < 0.0001). In the pathomorphism analysis of the LSTs, we found that the tubulovillous type contributed a lot (OR = 36.345; 95% CI 1.596–827.795; *p* = 0.024). Interestingly, LST-NG-FE decreased the risk of LSTs with polyps (OR = 0.283; 95% CI 0.081–0.985; *p* = 0.047) along with the low-grade LSTs (OR = 0.103; 95% CI 0.012–0.901; *p* = 0.040). The area under the curve (AUC) of the binary logistic model was 0.866 (95% CI 0.817-0.914; *p* < 0.001) (Figure 2(a)).

### 3.3. Colorectal Polyps in the Right Colon Increased the Malignant Risk of LSTs

It is clinically important to predict malignant histology before deciding the appropriate treatment for LSTs. The binary logistic model about factors associated with malignant histology in enrolled subjects was described in Supplemental Table 1. LSTs with simultaneous colorectal polyps were more likely to possess the advanced histology (OR 1.789; 95% CI 0.626-5.116). When LSTs were ≥4 cm and located in the hepatic flexure and descending colon, the risk of advanced histology positively increased. The AUC of the binary logistic model was 0.866 (95% CI 0.804-0.927; *p* < 0.001) (Figure 2(b)).

Factors associated with malignant risk of LSTs in the polyp group were described in Table 3. These patients were divided into two groups: 94 patients with nonmalignant histology and 25 patients with malignant histology. Most simultaneous polyps were less than 1 cm. The simultaneous polyps located in the right colon accounted for the most LSTs with malignant histology (*p* = 0.022). The LST size (*p* = 0.001) and morphology of LSTs (*p* = 0.012) were significantly different in the two groups. LST-G-Ms and LST-NG-PDs were more common in the malignant group. No significant differences were observed in gender, age, colorectal polyp size, the number of colorectal polyps, history of polys, locations, and pathological morphological type of LSTs between the two groups.

To demonstrate the significant association between malignant histology and clinicopathological parameters in the polyp group further, we used the binary logistic model with adjustments of additional confounders (Supplemental Table 2 and Table 4). The simultaneous polyps located in the right colon notably increased the risk of malignant histology (OR = 58.540; 95% CI 2.387–1435.933; *p* = 0.013). LST-NG-PD significantly increased the risk of malignant LSTs (OR = 20.982; 95% CI 1.726–255.121; *p* = 0.017). In terms of tumor size, when LSTs were ≥5 cm, the risk of malignant histology positively increased (OR = 37.604; 95% CI 1.213–1165.336; *p* = 0.038). Colorectal polyps' sizes and locations of LSTs showed no statistically significant correlation with LST malignant histology. The AUC of the binary logistic model was 0.921 (95% CI 0.874-0.969; *p* < 0.001) (Figure 2(c)).

## 4. Discussion

In this study, we found the risk of the polyp coexistence phenomenon increased in males and those between 50 and 75 years old, and LSTs with tubulovillous types statistically increased the risk of the polyp coexistence phenomenon. History of polyps was significantly related to the coexistence of LSTs and colorectal polyps. Hence, we could combine age, gender, tumor size, granular type, and history of polyps to predict the coexistence phenomenon. The AUC of the binary logistic model was 0.866 (95% CI 0.817-0.914; *p* < 0.001). LST-NG-PD (OR = 20.982, *p* = 0.017) and LSTs ≥ 5 cm (OR = 37.604, *p* = 0.038) notably increased the malignant risk of LSTs. When the simultaneous polyps were located in the right colon, the risk of malignant LSTs (OR = 58.540, *p* = 0.013) positively increased. Hence, we could combine locations of the simultaneous colorectal polyps along with sizes and morphological types of LSTs to predict malignant histology of LSTs. The AUC of the binary logistic model was 0.921 (95% CI 0.874-0.969; *p* < 0.001).

Our study showed LSTs localized mostly in the right colon, similar to those of previous studies in different countries [12–17]. LST-G-M was the most common subtype, which contradicted the data from Western countries [3]. It may be associated with different races. LST-NG-FE and low-grade dysplasia of LSTs significantly decreased the incidence of the coexistence of LST and colorectal polyps. As we all know, LST-NG-FE shows the low incidence of advanced LSTs. The result disclosed that LSTs with lower malignant risk may be associated with the low incidence of the simultaneous colorectal polyps. The carcinoma incidence rate and the submucosal invasion rate were reported to be higher in LST-NG-PD and LST-G-M, particularly in LST-NG-PD [3, 18]. In practice, LST-NG is more difficult to remove endoscopically than LST-G.

In our study, the size of simultaneous polyps was mostly <1 cm, which meant they were more likely to be nonadvanced. A recent study showed that there was no significant increase in the risk of colorectal cancer in patients with nonadvanced adenomas. Meanwhile, advanced adenomas and ≥1 cm serrated polyps increased the risk of CRC [19]. Hence, when colorectal polyps simultaneously presented with LSTs, endoscopists need to pay more attention to LSTs than to polyps. When adjusting the presence of simultaneous polyps with other risk factors, polyps in the right colon were the most important risk factor about the malignant risk of LSTs. A meta-analysis study revealed that the right-side location was not an independent risk factor for missed adenomas [20]. Therefore, polyps located in the right colon may be a reliable factor to predict the malignant risk of LSTs. LSTs with cancerous histology were reported associated with the adenoma recurrence after endoscopic therapy [21]. What is more, tumor seeding during colonoscopy is a possible cause for metachronous colorectal cancer [22]. Therefore, en bloc resection is essential if the malignant LSTs are suspected, which ensures the effectiveness of treatment, reduces the recurrence of polyps and LSTs, and potentially decreases the incidence of cancerous LST seeding [21–23]. But because of the small sample size, tumor seeding may be considered as controversial. Thus, when LSTs presented with the simultaneous colorectal polyps, we should manage stricter surveillance plans.

Moreover, endoscopic diagnosis and resection of LSTs are technically difficult. A recent study demonstrated that an AI model trained with an endoscopic video can differentiate diminutive adenomas from hyperplastic polyps with high accuracy. We may build an AI model to predict the coexistence phenomenon and malignant risk of LSTs with high sensitivity and specificity.

The strength of our study resides in the fact that our study was the first to present the risk factors of the coexistence of LSTs and colorectal polyps and stratify the risks of malignant histology by lesion size, subtypes, and polyp locations to provide a more solid basis for the treatment and follow-up strategies. Other than previous studies, our study revealed that simultaneous polyps in the right colon were the most important factor to predict the malignant risk of LSTs. Therefore, endoscopists or surgeons may perform more meticulous treatments to ensure the effectiveness of treatment, reduce the recurrence of polyps and LSTs, and potentially decrease the incidence of cancerous LST seeding. Several limitations to our study should be acknowledged. First, all of the subjects were from the same institution. Second, some bias cannot be avoided because of the limited number of patients. We need to enroll more subjects to verify our conclusions in more institutions in the near future. Third, during endoscopy, the simultaneous colorectal polyps presented benign features, but we had little information about the pathological features of the simultaneous colorectal polyps. Consequently, we did not evaluate the pathological association between LSTs and simultaneous colorectal polyps.

## 5. Conclusion

During endoscopy, we could combine age, gender, history of polyps, tumor size, and morphological type of LSTs to predict the coexistence phenomenon. When colorectal polyps simultaneously present with LSTs, we should pay more attention to the polyps' location, LSTs' subtypes, and size to predict the malignant risk of LSTs. Polyps located in the right colon were the strong predictor with regard to the malignant risk of LSTs. En bloc resection is essential if malignant LSTs are suspected, which ensures the effectiveness of treatment, reduces the recurrence of polyps and LSTs, and potentially decreases the incidence of cancerous LST seeding.

## Figures and Tables

**Table 1:** The baseline clinicopathological factors of the enrolled subjects.

Characteristics	*N*	Nonpolyps (*N* = 87)	Polyps (*N* = 119)	*p* value
Gender				0.005
Male	114	38	76	
Female	92	49	43	
Age (year)				0.239
≤44	9	7	2	
≤49	6	3	3	
50–75	169	70	99	
≥76	22	7	15	
Tumor diameter (cm)				0.001
<2	65	28	37	
<3	65	26	39	
<4	45	13	32	
<5	16	14	2	
≥5	15	6	9	
Location				0.439
Ileocecal valve	28	10	18	
Ascending colon	54	26	28	
Hepatic flexure	16	8	8	
Right transverse colon	12	4	8	
Left transverse colon	32	8	24	
Splenic flexure	2	1	1	
Descending colon	9	3	6	
Sigmoid colon	20	10	10	
Rectum	33	17	16	
Morphological type				0.070
G-H	27	12	15	
G-M	92	47	45	
NG-FE	56	20	36	
NG-PD	31	8	23	
Pathomorphism				0.013
Hyperplastic	14	5	9	
Tubular	148	58	90	
Tubulovillous	17	5	12	
Serrated adenoma	27	19	8	
Histological type				0.845
Hyperplastic	14	5	9	
Low grade	157	67	90	
High grade	23	11	12	
Carcinoma	11	3	8	
History of polyps				<0.0001
No	132	75	57	
Yes	74	12	62	

**Table 2:** Best-fitting binary logistic regression model for factors associated with the coexistence of LSTs and polyps.

	*N*	Case (%)	*p* value	OR	OR (95% CI)	
					Lower	Upper
Pathomorphism (tubulovillous)	17	12 (70.6%)	0.024	36.345	1.596	827.795
History of polyps (yes)	74	62 (83.8%)	<0.0001	13.245	4.962	35.359
Age (50–75 years)	169	99 (58.6%)	0.036	7.074	1.138	43.981
Tumor diameter (3–4 cm)	45	32 (71.1%)	0.003	5.768	1.808	18.398
Gender (male)	114	76 (66.7%)	0.047	2.138	1.009	4.530
Morphological type (NG-FE)	56	36 (64.3%)	0.047	0.283	0.081	0.985
Location (hepatic flexure)	16	8 (50.0%)	0.049	0.189	0.036	0.993
Histological type (low grade)	157	90 (57.3%)	0.040	0.103	0.012	0.901

**Table 3:** Factors associated with malignant LSTs in the polyp group.

	*N*	Nonmalignant (*N* = 94)	Malignant (*N* = 25)	*p* value
Gender				0.987
Male	76	60	16	
Female	43	34	9	
Age (year)				0.365
≤49 or ≥76	20	14	6	
50–75	99	80	19	
Location of colorectal polyps				0.022
Right colon	29	20	9	
Left colon	35	29	6	
Rectum	18	13	5	
Right colon+left colon	9	6	3	
Right colon+rectum	3	2	1	
Left colon+rectum	2	2	0	
3 sites	23	22	1	
Polyp size (cm)				0.973
<1	86	68	18	
≥1	33	26	7	
Number of polyps				0.926
≤2	80	63	17	
≥3	39	31	8	
LST size (cm)				0.001
<2	37	34	3	
<3	39	33	6	
<4	32	23	9	
<5	2	0	2	
≥5	9	4	5	
Location of LSTs				0.173
Ileocecal valve	17	16	1	
Ascending colon	30	25	5	
Hepatic flexure	8	5	3	
Right transverse colon	8	6	2	
Left transverse colon	23	19	4	
Splenic flexure	1	1	0	
Descending colon	6	3	3	
Sigmoid colon	10	9	1	
Rectum	16	10	6	
Morphological type				0.012
G-H	15	13	2	
G-M	45	30	15	
NG-FE	36	34	2	
NG-PD	23	17	6	
Pathomorphism				0.113
Hyperplastic	9	9	0	
Tubular	90	72	18	
Tubulovillous	12	7	5	
Serrated adenoma	8	6	2	
History of polyps				0.362
No	57	43	14	
Yes	62	51	11	

**Table 4:** Best-fitting binary logistic regression model for factors associated with malignant LSTs in the polyp group.

	*N*	Case (%)	*p* value	OR	OR(95% CI)	
					Lower	Upper
Location of colorectal polyps (right colon)	29	9 (30.0%)	0.013	58.540	2.387	1435.933
Morphological type of LSTs (NG-PD)	23	6 (26.1%)	0.017	20.982	1.726	255.121
Tumor diameter (≥5 cm)	9	5 (55.6%)	0.038	37.604	1.213	1165.336

## Data Availability

The authors declare that they agree to provide data.

## Supplementary Materials

Supplementary Materials Supplemental Table 1: binary logistic regression model for factors associated with malignant LSTs. Supplemental Table 2: binary logistic regression model for factors associated with malignant LSTs in polyps group.
