# Supplementary material for: The Coexistence of Colorectal Polyps in the Right Colon Increases the Malignant Risk of Laterally Spreading Tumors
Source: Gastroenterol Res Pract. 2020 Apr 14;2020:3180420. doi: 10.1155/2020/3180420 (PMC7178507; doi:10.1155/2020/3180420)
Supplement: Supplementary Materials — Supplemental Table 1: binary logistic regression model for factors associated with malignant LSTs. Supplemental Table 2: binary logistic regression model for factors associated with malignant LSTs in polyps group. [file 3180420.f1.doc]

**Supplemental Table 1.** Binary logistic regression model for factors associated with malignant LSTs

|  | Non-Malignant | | Malignant | *p* value | OR | OR（95% CI） | |
| --- | --- | --- | --- | --- | --- | --- | --- |
|  |  |  |  |  |  | Lower | Upper |
| Gender(Males) | | 91 | 23 | 0.665 | 1.226 | 0.487 | 3.081 |
| Age (year) | |  |  |  |  |  |  |
| ≤49 | | 6 | 0 | 0.999 | - | - | - |
| 50–75 | | 140 | 28 | 0.824 | 0.795 | 0.105 | 6.03 |
| ≥76 | | 14 | 8 | 0.961 | 0.947 | 0.107 | 8.364 |
| Tumor diameter (cm) | |  |  |  |  |  |  |
| <3 | | 58 | 7 | 0.926 | 0.933 | 0.213 | 4.092 |
| <4 | | 35 | 12 | 0.118 | 3.233 | 0.742 | 14.095 |
| <5 | | 10 | 6 | 0.01 | 10.675 | 1.783 | 63.926 |
| ≥5 | | 6 | 9 | 0.002 | 13.832 | 2.574 | 74.322 |
| Location | |  |  |  |  |  |  |
| Ascending colon | | 48 | 6 | 0.292 | 2.587 | 0.441 | 15.174 |
| Hepatic flexure | | 11 | 5 | 0.006 | 16.525 | 2.222 | 122.866 |
| Right transverse colon | | 9 | 3 | 0.097 | 6.496 | 0.713 | 59.155 |
| Left transverse colon | | 28 | 4 | 0.496 | 1.946 | 0.286 | 13.255 |
| Splenic flexure | | 2 | 0 | 0.999 | - | - | - |
| Descending colon | | 6 | 3 | 0.035 | 11.949 | 1.196 | 119.356 |
| Sigmoid colon | | 18 | 2 | 0.968 | 0.957 | 0.115 | 7.982 |
| Rectum | | 21 | 12 | 0.072 | 4.361 | 0.878 | 21.658 |
| Morphological type | |  |  |  |  |  |  |
| G-M | | 70 | 24 | 0.457 | 1.788 | 0.387 | 8.258 |
| NG-FE | | 53 | 3 | 0.182 | 0.275 | 0.041 | 1.831 |
| NG-PD | | 23 | 8 | 0.389 | 2.19 | 0.368 | 13.035 |
| Pathomorphism | |  |  |  |  |  |  |
| Tubular | | 118 | 30 | 0.998 | - | - | - |
| Tubulovillous | | 11 | 6 | 0.998 | - | - | - |
| Sessile | | 25 | 2 | 0.998 | - | - | - |
| Simultaneous polyps(Yes) | | 95 | 24 | 0.278 | 1.789 | 0.626 | 5.116 |

**Supplemental Table 2.** Binary logistic regression model for factors associated with malignant LSTs in polyps group

|  | Non-Malignant | Malignant | *p* value | OR | 95%CI | |
| --- | --- | --- | --- | --- | --- | --- |
|  |  |  |  |  | Lower | Upper |
| Location of colorectal polyps |  |  |  |  |  |  |
| Right colon | 20 | 9 | 0.013 | 58.54 | 2.387 | 1435.933 |
| Left colon | 29 | 6 | 0.431 | 3.147 | 0.181 | 54.754 |
| Rectum | 13 | 5 | 0.132 | 11.958 | 0.474 | 301.597 |
| Right colon+Left colon | 6 | 3 | 0.319 | 4.704 | 0.224 | 98.909 |
| Right colon+Rectum | 2 | 1 | 0.253 | 11.577 | 0.174 | 772.124 |
| Left colon+Rectum | 2 | 0 | 0.999 | 0 | 0 | - |
| Polyp size(≥1cm) | 26 | 7 | 0.121 | 3.62 | 0.713 | 18.369 |
| LST size(cm) |  |  |  |  |  |  |
| <3 | 33 | 6 | 0.437 | 0.42 | 0.047 | 3.746 |
| <4 | 23 | 9 | 0.279 | 3.728 | 0.345 | 40.293 |
| <5 | 0 | 2 | 0.999 | - | - | - |
| ≥5 | 4 | 5 | 0.038 | 37.604 | 1.213 | 1165.336 |
| Location of LSTs |  |  |  |  |  |  |
| Ascending colon | 25 | 5 | 0.998 | - | - | - |
| Hepatic flexure | 5 | 3 | 0.997 | - | - | - |
| Right transverse colon | 6 | 2 | 0.998 | - | - | - |
| Left transverse colon | 19 | 4 | 0.998 | - | - | - |
| Splenic flexure | 1 | 0 | 1 | - | - | - |
| Descending colon | 3 | 3 | 0.997 | - | - | - |
| Sigmoid colon | 9 | 1 | 0.998 | - | - | - |
| Rectum | 10 | 6 | 0.998 | - | - | - |
| Morphological type |  |  |  |  |  |  |
| G-H | 13 | 2 | 0.202 | 5.393 | 0.406 | 71.584 |
| G-M | 30 | 15 | 0.052 | 11.724 | 0.982 | 140.004 |
| NG-PD | 17 | 6 | 0.017 | 20.982 | 1.726 | 255.121 |
